# An End-To-End Speech Recognition Model for the North Shaanxi Dialect: Design and Evaluation

**DOI:** 10.3390/s25020341

**Published:** 2025-01-09

**Authors:** Yi Qin, Feifan Yu

**Affiliations:** 1 College of Computer Science & Technology, Xi’an University of Science and Technology, Xi’an 710054, China; 2 SHCCIG Yubei Coal Industry Co., Ltd., Xi’an 710900, China; 13310987687@163.com

**Keywords:** dialect speech recognition, coal mining industry, end to end, Conformer model, Transformer model, Connectionist Temporal Classification (CTC)

## Abstract

The coal mining industry in Northern Shaanxi is robust, with a prevalent use of the local dialect, known as “Shapu”, characterized by a distinct Northern Shaanxi accent. This study addresses the practical need for speech recognition in this dialect. We propose an end-to-end speech recognition model for the North Shaanxi dialect, leveraging the Conformer architecture. To tailor the model to the coal mining context, we developed a specialized corpus reflecting the phonetic characteristics of the dialect and its usage in the industry. We investigated feature extraction techniques suitable for the North Shaanxi dialect, focusing on the unique pronunciation of initial consonants and vowels. A preprocessing module was designed to accommodate the dialect’s rapid speech tempo and polyphonic nature, enhancing recognition performance. To enhance the decoder’s text generation capability, we replaced the Conformer decoder with a Transformer architecture. Additionally, to mitigate the computational demands of the model, we incorporated Connectionist Temporal Classification (CTC) joint training for optimization. The experimental results on our self-established voice dataset for the Northern Shaanxi coal mining industry demonstrate that the proposed Conformer–Transformer–CTC model achieves a 9.2% and 10.3% reduction in the word error rate compared to the standalone Conformer and Transformer models, respectively, confirming the advancement of our method. The next step will involve researching how to improve the performance of dialect speech recognition by integrating external language models and extracting pronunciation features of different dialects, thereby achieving better recognition results.

## 1. Introduction

In recent years, the rapid advancement of artificial intelligence technology has spurred the further development of intelligent coal mine construction [1]. The introduction of policies, such as the “Intelligent Coal Mine Guide (2021 Edition)” and the “Trial Measures for the Acceptance Management of Intelligent Demonstration Coal Mines”, has underscored the growing necessity of establishing intelligent coal mines that leverage related artificial intelligence technologies [2,3]. Given the unique characteristics of the coal industry, developing an intelligent coal mine that includes a voice interaction system tailored to the sector is crucial for ensuring the safety of coal mine production.

In the coal mine scenario, automatic speech recognition (ASR) systems aimed at handling dialect accents may face the following specific requirements.

High Accuracy. The ASR system needs to be able to accurately recognize speech with local characteristics, even under the influence of background noise and the miners’ dialect accents.

Strong Robustness. The system should be able to work stably in a coal mine environment, with interference from machine noise, blasting sounds, and other work-related noises.

Dialect Adaptability. The ASR (automatic speech recognition) system needs to be able to adapt to and recognize specific regional dialects, which may require the collection and training of dialect datasets.

Real-time Processing. In emergency situations, the ASR system needs to be capable of processing and responding to voice commands in real-time to enable quick decision-making.

Noise Suppression. The system should have effective noise suppression technology to reduce the interference of environmental noise in speech recognition.

Far-field Recognition Capability. Due to the special nature of the coal mine environment, the ASR system needs to have far-field speech recognition capability, allowing it to accurately capture speech from a greater distance.

Multi-speaker Recognition. The system should be able to handle situations with multiple speakers, distinguishing between the voices of different speakers and accurately recognizing each one.

Contextual Understanding. The ASR system should not only recognize speech but also understand the specific context of the miner’s instructions or reports to execute tasks more accurately.

Ease of Use and Wearability. Considering the working conditions of miners, the ASR system should be user friendly and wearable, without hindering the normal work of the miners.

Safety and Privacy Protection. The system needs to ensure the security of collecting and processing voice data and the privacy protection of the miners.

Diversity of Training Data. To enhance the system’s generalization capability, the training data should include a variety of dialects and accents, as well as different speaking rates and styles.

User Customization. The system may need to offer customization options to allow miners to adjust the system settings according to their own accents and speaking habits.

Northern Shaanxi, one of the most coal-rich regions in China, is at the forefront of producing and managing the production environment, conferences, dispatching, and command operations within the coal industry. The integration of a voice interaction system that accommodates the local dialect is vital for enhancing communication efficiency and safety in these contexts.

Management personnel in the coal mining industry predominantly communicate using the North Shaanxi dialect, commonly referred to as “Shaanxi Pu,” which is characterized by a distinct Northern Shaanxi accent. The dialect serves not only as a cultural emblem but also as a carrier of traditional cultural heritage. Consequently, it is imperative to compile a corpus of the North Shaanxi dialect and to develop speech recognition capabilities tailored to the coal mining industry. This initiative is vital for both preserving the regional culture and enhancing operational efficiency within the sector.

To address the challenge of dialect recognition within the field of speech recognition, initial research efforts by scholars involved adapting traditional speech recognition models for dialect recognition purposes. This included the application of linear predictive coding (LPC), dynamic time warping (DTW), and hidden Markov models (HMMs) as technical frameworks for dialect identification. Furthermore, the integration of Gaussian Mixture Model (GMM) technology in speech modeling has notably enhanced recognition rates. For instance, studies [4,5,6] have utilized the GMM to develop dialect recognition systems for the Mongolian dialect, Chongqing dialect, and the Shuozhou dialect in Shanxi Province.

Due to the reliance of traditional dialect recognition methods on extensive corpora and manual annotation, these approaches incur high costs and often yield only moderate recognition performance. Moreover, the advent of deep learning has transformed the landscape of speech recognition technology.

Technology, particularly deep learning, not only streamlines the process of speech recognition but also markedly enhances recognition accuracy. For instance, the study in the literature [7] developed an end-to-end Listen, Attend, and Spell (LAS) model for Tujia speech recognition, incorporating a multi-head attention mechanism to boost the accuracy of Tujia dialect recognition. In the document by [8], an end-to-end dialect speech recognition method based on transfer learning is introduced, which leverages shared feature extraction to enhance the recognition performance of low-resource dialects. The document by [9] presents an end-to-end speech recognition system that integrates a multi-head self-attention mechanism with a residual network (ResNet) and a bidirectional long short-term memory network (Bi-LSTM), significantly improving the recognition of Jiangxi and Hakka dialects. Nonetheless, these models could benefit from further enhancements in incorporating contextual semantic information and capturing positional details.

Currently, end-to-end (E2E) speech recognition technology has yielded substantial research outcomes [10]. Architectures such as Recurrent Neural Networks (RNNs) [11], Convolutional Neural Networks (CNNs) [12,13], self-attention-based Transformer networks [14], and the Conformer [15] have emerged as prominent backbone structures for automatic speech recognition (ASR) models, garnering significant attention. However, the Conformer decoder exhibits limited text generation capabilities and the Transformer model is computationally intensive, incurs substantial memory costs, and has a weaker ability to capture local features.

Addressing these limitations, this study introduces a Conformer–Transformer–CTC (Connectionist Temporal Classification) fusion approach for dialectal speech recognition systems. The proposed method leverages the audio modeling prowess of the Conformer as the encoder, utilizes the Transformer for text generation as the decoder, and harnesses the flexible alignment capabilities of CTC to construct an end-to-end dialect speech recognition model, thereby enhancing speech recognition accuracy.

The end-to-end speech recognition model proposed in this paper for the North Shaanxi dialect demonstrates innovation in terms of customization for specific dialects, end-to-end architecture, adaptive feature extraction, and robustness against environmental noise.

## 2. Related Work

The end-to-end speech recognition process entails the consolidation of acoustic, pronunciation, and linguistic factors within a single deep neural network (DNN) to streamline the modeling process and achieve direct mapping from speech input to text output [16,17]. As deep learning techniques gain widespread application across various domains, end-to-end speech recognition models have demonstrated superior recognition performance compared to traditional speech recognition models, garnering increasing interest and attention [18,19].

Currently, the prevalent end-to-end methods include (a) Connectionist Temporal Classification (CTC) [20], which computes the model’s loss and optimizes it using forward and backward algorithms [21]. A study by [22] further introduces intermediate CTC loss to regularize CTC training and enhance speech recognition performance. (b) A Recurrent Neural Network Transducer (RNN-T) [23] is suitable for streaming speech recognition, as it can recall past information. (c) The encoder–decoder architecture with an attention mechanism [24] has garnered significant attention and research. In recent years, the diverse modeling approaches in end-to-end automatic speech recognition (ASR) systems have sparked interest in developing hybrid methods to leverage their complementary strengths for speech recognition [25]. For instance, the research in [26] combined CTC with the Transformer decoder to achieve higher recognition accuracy. The study in [27] integrated the self-attention mechanism and the multi-layer perceptron module into dual branches within the model, yielding exceptional recognition performance.

In essence, the integration of the strengths of different architectures has demonstrated potential for enhancing speech recognition performance in recent research [28]. In light of this, this study harnesses the benefits of end-to-end models to construct a speech recognition system for the North Shaanxi dialect, aiming to improve recognition performance.

## 3. Method

### 3.1. Corpora Establishment

**Figure 1:**
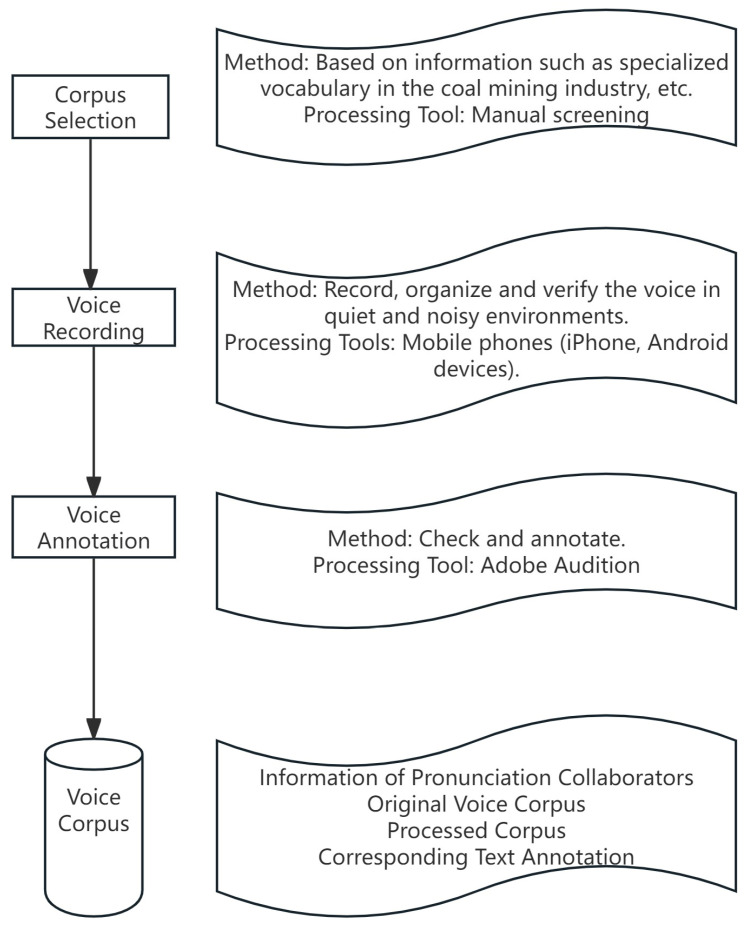
The construction process of dialect corpora.

Currently, there is a significant lack of openly accessible corpora concerning the dialect used within the coal mining sector in Northern Shaanxi. To facilitate research in the realm of speech recognition for the North Shaanxi dialect, this study has successfully compiled a specialized dialect corpus for the Northern Shaanxi coal mining industry. The methodology employed in constructing this corpus is detailed in Figure 1.

The initial phase involves the careful selection of speech materials from the region. By analyzing the distinctive features of the North Shaanxi dialect, which are summarized in Table 1, the corpus was compiled. The selection process was guided by the textual content of coal mine dispatch logs, industry-specific terminology from the coal mining sector, and relevant industrial texts. Subsequently, recorded transcripts were created based on this selected material.

The recording protocol adopted in this study involves the collection of dialect data by 20 volunteers. The recordings are primarily based on the text from the Northern Shaanxi coal mine scene-specific dialect dataset. Among the 20 volunteers, there are 13 males and 7 females, with ages ranging from 18 to 40 years. All participants are native to Northern Shaanxi, and the dialects recorded are exclusively of the Northern Shaanxi variety. The resultant dataset is detailed in Table 2.

To guarantee the quantity and integrity of the data, this study employs professional recording equipment to capture the audio. The recordings are saved in WAV format with a 16 kHz sampling rate. Subsequent to recording, the audio files are meticulously checked and annotated using Adobe Audition.

In the final phase of data preparation, all recorded data, including participant information, the original phonetic corpus, and the annotated and processed corpus along with the corresponding text annotations, are systematically organized and stored within a unified corpus repository. This structured approach ensures that the dataset is both comprehensive and accessible for further analysis and use in speech recognition model training.

### 3.2. Conformer–Transformer–CTC Model Structure

**Figure 2:**
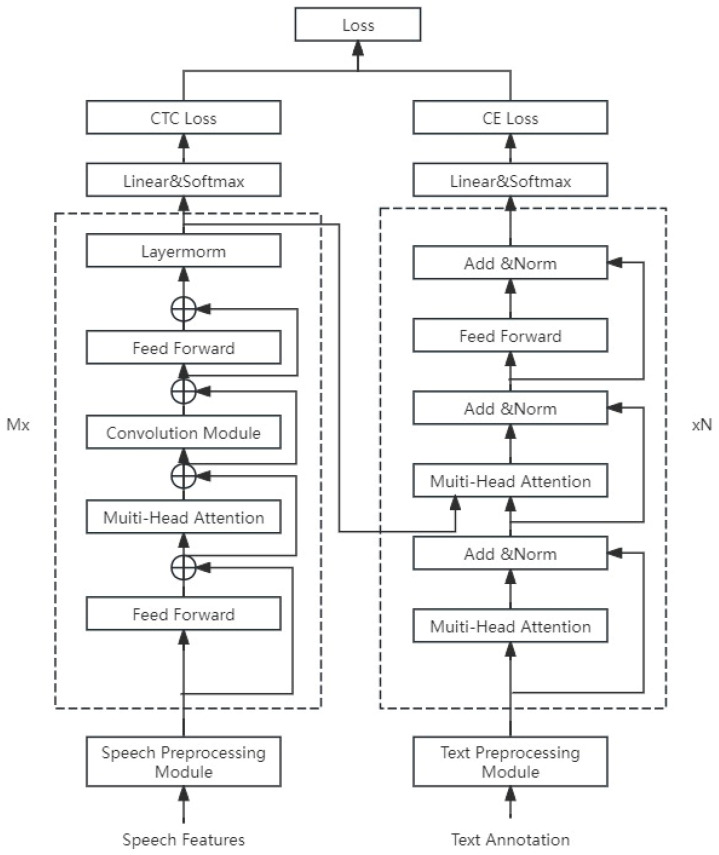
End-to-end dialect Conformer–Transformer–CTC speech recognition system.

To achieve a better dialect recognition rate in dialect speech recognition models, this paper establishes a Conformer–Transformer–CTC speech recognition system, which includes preprocessing modules (speech preprocessing module, text preprocessing module) and an encoder–decoder (the encoder uses a Conformer, and the decoder uses a combination of a Transformer and CTC for joint decoding). Figure 2 shows the end-to-end dialect Conformer–Transformer–CTC speech recognition system.

#### 3.2.1. Preprocessing Module

**Figure 3:**
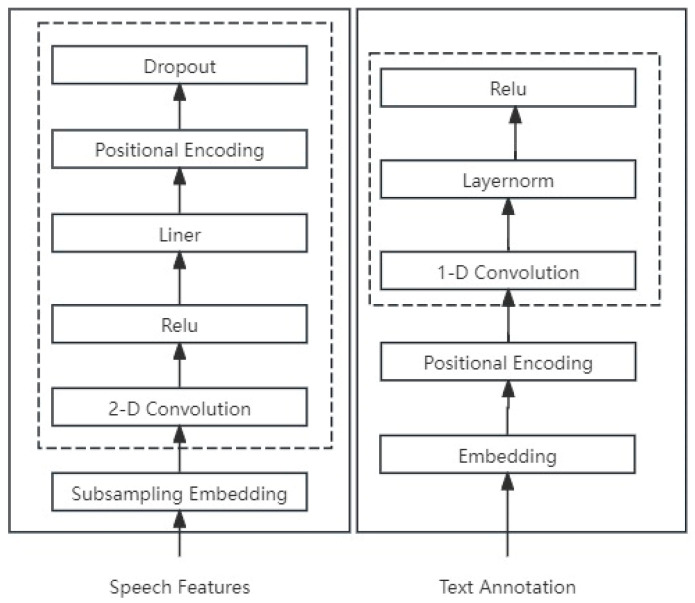
Preprocessing module.

The preprocessing module includes a speech preprocessing module and a text preprocessing module, as shown in Figure 3.

The speech processioning module developed in this study comprises a downsampling module (Sub-sampling Embedding), a convolution module, and position coding (position encoding).

Firstly, the speech features are downsampled. Downsampling is a commonly used technique in data processing that reduces the complexity of data by decreasing its temporal resolution. In the field of speech processing, downsampling can help models better handle issues such as significant variations in speech duration or rapid speaking rates caused by dialects.

When processing dialectal speech, due to the characteristics of dialects, there may be significant variations in the duration of speech, or the speaker may talk at a very fast pace. These characteristics can make it difficult for the model to accurately recognize and process the speech signal. By downsampling, we can reduce the temporal resolution of the speech signal, thereby enabling the model to more effectively handle rapidly changing speech features.

Acoustic features in dialectal speech may exhibit spatiotemporal correlations. This means that in dialectal speech recognition, acoustic features not only have continuity over time but also spatial correlations. Using downsampling techniques, these features can be effectively captured, thereby improving the accuracy and efficiency of speech recognition.

Therefore, the following convolutional module, which includes a 2D convolution layer and a ReLU activation layer, is used to capture the patterns of acoustic features in both time and frequency dimensions, learning the local features within dialectal speech. At the same time, since dialectal speech may have acoustic features that differ from standard languages, the ReLU activation function can enhance the model’s expressive power for these features.

Following this, a linear layer is employed to extract dialect-specific acoustic features and patterns, yielding a more tailored feature representation. Additionally, fixed position coding, utilizing sine and cosine functions, is applied to better comprehend the sequential distribution and structure of dialectal speech features, as detailed in Formulas (1) and (2).

Conclusively, the application of Dropout operations involves the random suppression of certain features, which decreases the model’s reliance on individual features and, in turn, strengthens its robustness and generalization capabilities.

(1) $$PE_{\left(pos,2i\right)}=\sin(pos/10000^{2i/d_{\mathrm{mod}el}})$$

(2) $$PE_{\left(pos,2i+1\right)}=\cos(pos/10000^{2i/d_{\mathrm{mod}el}})$$

*PE* refers to the position encoding matrix, where pos denotes the specific position of the current character in the sequence, *i* represents the *i*-th dimension of the character vector, and $d_{\mathrm{mod}el}$ indicates the dimension size of the character vector.

Through the aforementioned processes, the speech processioning module facilitates dimensional reduction, feature transformation, and position modeling for dialectal speech. This results in a feature representation with enhanced discriminating power, thereby improving the performance and accuracy of dialect recognition. Utilizing this speech processioning module enables the model to more effectively adapt to the characteristics of dialectal speech and to extract dialect-specific acoustic features and patterns.

The text processioning module developed in this paper includes an embedding layer, position coding, and convolution modules.

Initially, the embedding layer is employed to convert text labels into dense vector representations, capturing the semantic relationships between words. Subsequently, the same position coding technique used in the speech preprocessing module is applied to encode the positional information of the words. Following this, a 1D convolutional layer is utilized to extract implicit positional information and to capture more nuanced local semantic details. Layer normalization is applied to normalize the model’s output at this stage. Finally, the ReLU activation function is introduced to incorporate non-linearities, thereby enhancing the model’s representational capacity.

By undergoing this text processing pipeline, the speech recognition system can enhance its comprehension and expression of textual information, thereby improving the overall performance of speech recognition.

#### 3.2.2. Codec

**Figure 4:**
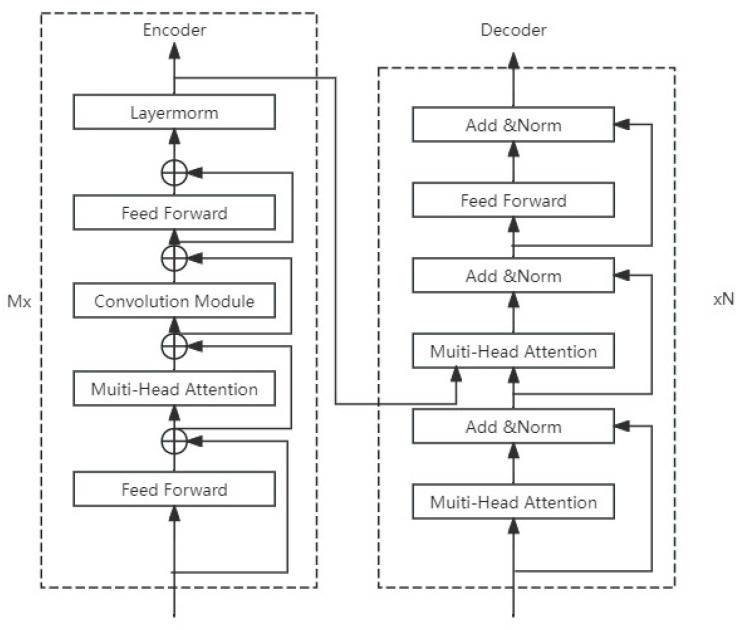
Codec structure.

The advantage of the Conformer architecture as an encoder lies in its ability to process both time-domain and frequency-domain features of the audio signal. This dual processing capability allows the Conformer to yield a rich audio representation, enhancing its understanding of the input audio signal and providing more informative features for the subsequent decoder stage. Nevertheless, the Conformer structure exhibits limited text generation capabilities within its decoder. To address this, the present study employs a Transformer-based decoder. The self-attention mechanism inherent in the Transformer is adept at managing long-range dependencies, which allows the decoder to take into account the global context when generating text. This results in the production of accurate and coherent textual outputs. The proposed architecture is depicted in Figure 4.

- Encoder

The Conformer model primarily consists of four key modules: the first feedforward module (feedforward), the multi-head attention module (multi-head self-attention), the convolutional module (convolution module), and the second feedforward module. The Conformer calculates the output $h_{i}$ for the input vector $x_{i}$ of the i-th encoder, and the equation is as follows:

(3) $${\tilde{x}}_{i}=x_{i}+\frac{1}{2}FFN(x_{i})$$

(4) $$x_{i}^{\prime }={\tilde{x}}_{i}+MHSA({\tilde{x}}_{i})$$

(5) $$x_{i}^{\prime \prime }=x_{i}^{\prime }+Conv(x_{i}^{\prime })$$

(6) $$y_{i}=Layernorm(x_{i}^{\prime \prime }+\frac{1}{2}FFN(x_{i}^{\prime \prime }))$$

Among the components, FFN denotes the feedforward module. “First” signifies the initial feedforward module, succeeded by the “Second” feedforward module. MHSA stands for the multi-head self-attention module, while “Conv” is an abbreviation for the convolution module. “Layernorm” represents layer normalization. Each of these modules incorporates a residual connection to enhance the flow of gradients and stabilize training.

- Decoder

$x=(x_{1},x_{2},\ldots x_{n})$ represents the input sequence, $h=(h_{1},h_{2},\ldots h_{t})$ represents the advanced sequence, $y=(y_{1},y_{2},\ldots y_{i})$ is the output sequence, and the probability of encoding and decoding is as follows:

(7) $$P(y|x)=\prod_{t=1}^{T}P(y_{t}|h,y<t)$$

At each time t, calculate the conditional dependency of the output on the encoder features h through the attention mechanism. The attention mechanism is a function of the hidden state of the current decoder and the encoder output features, which are compressed into a context vector. $v^{t}$, b, $W_{h}$, and $W_{d}$ are the learning parameters. The attention distribution is obtained by normalizing with softmax as follows:

(8) $$\alpha_{t}=soft\max(v^{t}\tanh(W_{h}h_{i}+W_{d}d_{t}+b)$$

Using $\alpha_{t}$ and hiding states $h_{i}$, the corresponding context direction is obtained using weighted sums as follows:

(9) $$c_{t}=\sum_{i=1}^{K}\alpha_{t}h_{i}$$

Finally, a Transformer is used as the decoder, and the training loss function is defined as follows:

(10) $$Att_{loss}=-\ln(P(y|x))$$

#### 3.2.3. CTC Auxiliary Training

Methods for improving speech recognition assistance tasks were investigated by assuming different levels of learning representations at different levels [29]. In this paper, the CTC objective function is integrated into the Conformer–Transformer fusion model. End-to-end training through CTC learning sequence-to-sequence mapping without explicit alignment reduces irregular alignment of Attention-based Encoder–Decoder (AED) models for better performance.

During training, the model consists of three parts, the Conformer encoder, the Transformer decoder, and the CTC decoder. The end-to-end dialect speech recognition training is as follows:

(11) h=Encoder(x)

(12) P(y|x)=Decoder(h)

The output of the encoder is used to calculate the CTC loss. Let the training set be S, and then the CTC loss function is as follows:

(13) $$CTC_{loss}=-\sum_{(X,Y)\in S}\ln P(y|x)$$

Combining the CTC loss and the decoder loss facilitates the convergence of the decoder while enabling the hybrid model to exploit the label dependence. Since CTC is used to assist the decoder alignment, CTC is less weighted in the fusion. The total loss function is defined as the weighted sum of CTC and the decoder loss as follows:

(14) $$T_{loss}(x,y)=\lambda CTC_{loss}(x,y)+(1-\lambda )Att_{loss}(x,y)$$

Among them, λ∈[0,1] is used to measure the importance of the CTC loss and the decoder loss. x represents speech features, while y represents text annotations.

### 3.3. Analysis of the Conformer–Transformer–CTC Model

In the research on end-to-end speech recognition models for the North Shaanxi dialect, the Conformer–Transformer–CTC model includes the following special designs for the unique coal mine scenario.

Dialect Feature Embedding. The model will include an acoustic feature embedding layer specific to the Shaanxi Northern dialect to better capture the tonal and pronunciation characteristics of the dialect.

Multilingual Pre-training. The model may use a dataset that includes standard Mandarin and the North Shaanxi dialect during the pre-training phase so that the model can learn the differences between the two languages.

Dialect Adaptation Layer. In the decoder part of the model, a dialect adaptation layer may be added to adjust the probability distribution of the model’s output in order to adapt to the grammatical and lexical characteristics of the North Shaanxi dialect.

Attention Mechanism Optimization. The attention mechanism should be enhanced to better handle long-term dependencies and specific phonetic liaisons commonly found in dialects.

Dialect Data Augmentation. The diversity of dialect training data should be increased using methods such as phoneme substitution, time stretching, and noise addition to improve the model’s generalization capabilities.

Custom Loss Function. A loss function that better reflects the difficulty of dialect recognition should be designed, such as assigning higher weights to phonemes that are unique to the dialect.

Joint Optimization of Acoustic and Language Models. During the training process, optimize both the acoustic model and the language model simultaneously to ensure they work in concert, thereby improving the accuracy of speech recognition.

In these special designs, the use of downsampling processing can reduce data dimensionality and computational complexity while retaining the main energy and key spectral information of the speech signal as much as possible. The frequency range of speech typically spans from 20 Hz to 20 kHz. If a 50% downsampling is performed, the frequency range of the speech will be reduced from the original 20 Hz–20 kHz to 10 Hz–10 kHz. In the context of speech recognition for the North Shaanxi dialect, the frequency components of the speech signal are very rich, necessitating the use of an anti-aliasing filter to prevent aliasing effects.

## 4. For Experimental Validation

### 4.1. Experimental Indicators

The experimental results are evaluated on a self-built dialectal speech dataset, using the word error rate (WER) as the algorithmic evaluation metric. The word error rate (WER) is an important evaluation indicator used to measure the accuracy of speech recognition systems. By calculating the number of substitution, deletion, and insertion errors between the recognition results and the reference text and dividing this by the total number of words in the reference text, the WER value can be obtained. This calculation method not only provides a comprehensive assessment of the performance of speech recognition systems but also offers important references for further optimization and improvement. The calculation method is shown in Equation (15) as follows:

(15) $$WER=\frac{I+D+S}{T}\times 100\%$$

where *I* represents the number of miswords added, *D* refers to the excluded words, *T* refers to the total word of the whole sentence, and *S* refers to the number of words that are replaced. A smaller *WER* value indicates a better identification effect.

### 4.2. Experimental Configuration

The hardware configuration used for the experiment is an Intel (R) Xeon (R) Gold 6330 processor (Intel Corporation, Santa Clara, CA, USA) with 32 GB of running memory and an NVIDIA GeForce RTX 3090 GPU (NVIDIA Corporation, Santa Clara, CA, USA). The software environment used is Anaconda 3 and Python 3.8 environments based on PaddlePaddle 2.4.1 deep learning framework under a Ubuntu 20.04.2 LTS operating system. The model was trained simultaneously with the Adam optimizer and the learning rate adaptive change strategy [30].

### 4.3. Experimental Data

The corpus of the North Shaanxi dialect used in the model includes the prescribed exclusive vocabulary of the coal mine industry, self-selected vocabulary, common sayings, coal mine dispatching calls, coal mine reports, and other related corpora, with a total of 9770 sentences and a total length of 25 h. The types of the corpus include vocabulary and grammar, oral culture, dialect dialogue, and dialect narration, as shown in Table 3, which are part of the data content of the corpus of the North Shaanxi dialect.

### 4.4. Experimental Results and Analysis

In this paper, different experiments are set up from the following three aspects, feature extraction, parameter tuning, and recognition rate (comparison experiment), which verify the influence of feature extraction technology and model parameters on the recognition rate, and the recognition rate of this model is better than the mainstream model. The details are as follows.

#### 4.4.1. Feature Extraction Experiment

Due to the phonetic characteristics of consonants and vowels in dialects, it is difficult to achieve optimal performance in the North Shaanxi dialect corpus using a single speech feature extraction technique. Therefore, based on the characteristics of consonants and vowels in the North Shaanxi dialect, multiple speech features are extracted to examine the impact of different feature extraction techniques on the performance of dialect speech recognition. The results are shown in Table 4.

Considering the above analysis, the root cause can be attributed to the differences in the ability of different characteristics and the information expression mode. The MFCC features are relatively weak, but the combination with other features can provide some complementary information. The combination of different features can provide a more comprehensive and mainly rich audio feature representation, thus improving the performance of the speech recognition system.

#### 4.4.2. Parameter Tuning Experiment

Since the self-built North Shaanxi dialect dataset cannot fully match the end-to-end model of depth, it is necessary to tune the model hyperparameters to perform the best results as far as possible. In this paper, there are several key parameters of the model, such as the depth and width (layer number and dimension) of the encoder (Conformer module), the multiple heads and dimensions of the self-attention mechanism, and the size of the deep convolution kernel. By selecting these parameters, the best model parameters are suitable for the dataset of the North Shaanxi dialect.

- Number of Conformer modules

For this, adjust the number and dimension of Conformer modules, set the number of Conformer modules according to 16, 12, and 8, and set the corresponding dimensions according to 1024, 2048, and 4096; the other parameters remain unchanged.

The experimental results in Table 5 show the influence of the number of encoders (encode number) and the encoder dimension (encoder dimension) in different groups on the performance. In the WER index, group 3 achieves the optimal effect. The dimension of the encoder was increased to 2048, providing a larger parameter space to capture the complex features of the audio. Higher encoder dimensions can better represent audio data and learn a richer feature representation, thus improving the accuracy of speech recognition. The poor performance in group 1 is due to the larger number of encoders, resulting in excessive model parameters and increasing the risk of overfitting. The performance in group 3 is also poor, probably due to the high dimension of the encoder, which makes the model too complex to fully learn and generalize. Therefore, we find the optimal parameters to achieve balance, thus improving the performance of the speech recognition system.

- The self-attention module

For this, the multiple heads of the encoder self-attention module are set according to 2, 4, and 8, and the corresponding dimensions are set according to 512, 256, and 128; the other parameters remain unchanged. The self-attention in the corresponding decoder is also set consistently for experiments.

The experimental results in Table 6 show that the influence of self-attention multiple heads and each head dimension on speech recognition performance are interrelated, and in the WER index, group 1 achieves the optimal effect. In this group, the dimension per attention long head is high, allowing each head to better capture the key information in the input sequence, and the multiple heads are balanced with the dimension, thus reducing identification errors. Groups 2 and 3 performed poorly relative to group 1. As the number of self-attention multiple heads increases, the dimensionality of each head decreases. The lower head dimension may limit the expression ability of each head to the input sequence, causing the model to accurately learn key features.

- Convolution module

For this, the convolution kernel size is singular, and the experiments were conducted according to 3, 7, and 15; the other parameters are unchanged.

The experimental results in Table 7 indicate that group 1 had the best results. Generally speaking, the larger the convolution core, the larger the receptive field, the more information the network “sees”, and the better the obtained feature representation. However, in the current scale dialect, the use of a small convolutional kernel size can better capture local details, which may help to better extract the characteristics of local details. The large convolutional kernel instead blurs these detailed features, which makes group 1 better than the other two groups. At the same time, the larger the size, the larger the computation, and the greater the computational cost.

#### 4.4.3. Comparison Experiments

Using a self-built corpus, some mainstream end-to-end models, including WeNet (Conformer) and WeNet (Transformer), are identified. Through the results shown in Table 8, it can be seen that this model is better than other mainstream models.

The experimental results show that the Conformer end-to-end dialect recognition model improves the sequence generation ability by introducing Transformer and CTC while enhancing the ability to model long sequences and flexible alignments. At the same time, it shows that when the preprocessing module is not used, the recognition performance is lower than that of the preprocessing module. Through dimension reduction, feature transformation, and position modeling, the model is more adapted to the phonetic characteristics of the North Shaanxi dialect and better extracts the unique acoustic features and patterns of the dialect.

## 5. Conclusions and Outlook

This paper proposes an end-to-end dialect speech recognition model for the coal mining industry in Northern Shaanxi. The Conformer–Transformer–CTC fusion approach leverages the strengths of each component, including robust feature extraction, accurate sequence generation, and alignment flexibility. Through experimental analysis and comparison, it has been demonstrated that the dialect speech recognition model proposed in this paper is more suitable for the North Shaanxi dialect, resulting in a lower error rate and better generalization performance. The next step will involve researching how to effectively integrate external language models to enhance the performance of dialect speech recognition while simultaneously exploring how to extract more effective pronunciation features from different dialects to achieve better recognition results and to further expand into other official dialects.

## Figures and Tables

**Table 1:** Characteristics of the North Shaanxi dialect.

Vocabulary Characteristics	Grammatical Characteristics	Expression Habits
Retains many ancient Chinese vocabulary words	There are some special sentence structures	The dialect is rich in slang, proverbs, and idioms, possessing distinct local characteristics.
There are many unique dialectal vocabulary words	The use of particles is quite rich	When speaking, one tends to use rhetorical devices, such as hyperboles and metaphors.

**Table 2:** Self-built corpus of Northern Shaanxi pronunciation.

Dataset	The Number of People	Duration	The Number of Sentences
training set	15	21 h	8010
test set	5	2 h	760
development set	5	2 h	760

**Table 3:** Part of the North Shaanxi dialect dataset.

The Corpus Type	Chapter Name	Duration
Lexicon grammar	A proprietary vocabulary of the coal mining industry (specified vocabulary)	For 180 min and 32 s
	Choose a vocabulary	66 min and 13 s
	Grammar example sentence	70 min and 32 s
Oral culture	Common saying	30 min and 11 s
	Phrase	28 min and 36 s
Dialect dialogue	Daily dialogue	70 min and 32 s
	Coal mine scheduling call	123 min and 2 s
Dialect	Coal mine report	60 min and 45 s

**Table 4:** Keyword rates under different feature extraction techniques.

Phonetic Feature	Error Word Rate (WER%)
MFCC (Mel-frequency cepstral coefficients) feature	32.3
FBank (Filter Bank) feature	29.5
Log-Mels (Logarithmic Mel-frequency spectrogram) feature	30.2
MFCC + FBank feature	33.2
MFCC + Log-Mels feature	27.6
Fbank + Log-Mels feature	28.8
MFCC + FBank + Log-Mels features	29.4

**Table 5:** Conformer module change test results.

Group	Encoder Count	Encode Dimension	WER (%)
1	16	1024	31.5
2	12	2048	26.9
3	8	4096	20.4

**Table 6:** Attention module change test results.

Group	Attention Multiple Head Number	Each Head Dimension	WER (%)
1	2	512	26.9
2	4	256	28.8
3	8	128	29.6

**Table 7:** Convolution module change test results.

Group	Convolutional Kernel Size	WER (%)
1	3	26.9
2	7	27.8
3	15	28.6

**Table 8:** Recognition rates of the North Shaanxi dialect datasets on different models.

Model	Error Word Rate (WER%)
WeNet (Conformer)	36.1
WeNet (Transformer)	37.2
Transformer–CTC	34.5
Conformer–CTC	33.8
Ours	26.9
Ours (pretreatment module)	30.8

## Data Availability

Data are contained within the article.
